# Nanophotonic Design of Germanium‐Silicon Single‐Photon Detectors for Integrated Quantum Photonic Circuits

**DOI:** 10.1002/nap2.70188

**Published:** 2026-06-30

**Authors:** Kuldeep Singh, Ekaterina Ponizovskaya Devine, Md Sakibul Islam, Nitesh Singh, Wayesh Qarony

**Affiliations:** ^1^ Department of Electrical and Computer Engineering University of Central Florida Orlando Florida USA; ^2^ W&Wsens Devices Inc. Los Altos California USA; ^3^ Department of Physics University of Central Florida Orlando Florida USA; ^4^ CREOL, The College of Optics and Photonics University of Central Florida Orlando Florida USA

## Abstract

Scalable on‐chip quantum photonic systems require single‐photon detectors that simultaneously provide high detection efficiency, low timing jitter, high bandwidth, and low noise while remaining compatible with silicon photonics and room‐temperature operation. Germanium–silicon (Ge/Si) single‐photon avalanche detectors (SPADs) offer a promising CMOS‐compatible platform for telecom‐band photon detection; however, existing implementations often suffer from limited photon‐detection efficiency, excess timing jitter, and challenges in monolithic integration with quantum photonic circuits. Here, we present a nanophotonic design of waveguide‐integrated Ge/Si SPADs engineered to address these limitations through enhanced optical confinement, engineered germanium absorption, and optimized avalanche multiplication in silicon. Finite‐difference time‐domain (FDTD) and carrier‐transport simulations demonstrate efficient evanescent coupling from the silicon waveguide into the Ge absorber while spatially confining the highest electrostatic field within the silicon multiplication region. Under simulated operating conditions, the proposed devices exhibit near‐unity internal quantum efficiency, avalanche gain exceeding 10^6^, sub‐50 ps intrinsic temporal response, electrical bandwidths beyond 5 GHz, and dark current below 100 pA, corresponding to low projected dark‐count‐rate operation. These projected performance characteristics establish a promising pathway toward high‐fidelity telecom‐band on‐chip single‐photon detection and scalable room‐temperature integrated quantum photonic circuits for quantum information processing applications.

## Graphical abstract

Nanophotonic Ge/Si single‐photon avalanche detectors (SPADs) employing photonic‐crystal‐enhanced optical confinement and a separate absorption–multiplication (SAM) architecture enable near‐unity internal quantum efficiency, low dark current, sub‐50 ps temporal response, and efficient telecom‐band single‐photon detection. The CMOS‐compatible platform offers a scalable route toward room‐temperature integrated quantum photonic circuits for on‐chip quantum information processing.

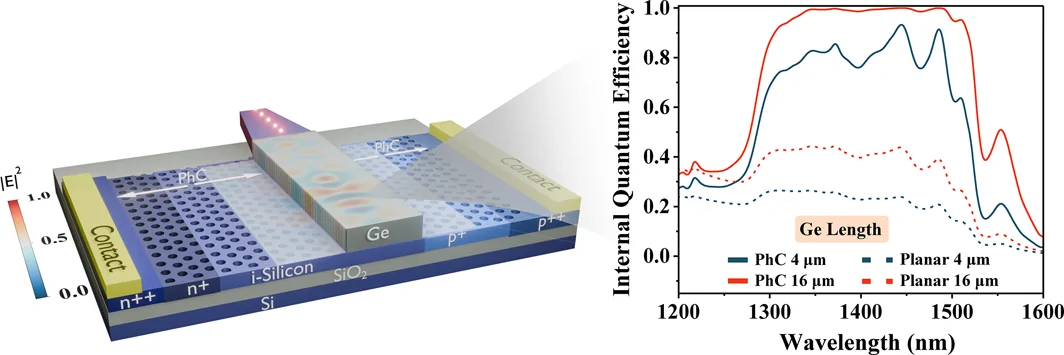

## 1 Introduction

Photonic quantum computing has emerged as a leading platform for scalable quantum information processing, communication, and distributed quantum networking [1, 2, 3, 4, 5, 6]. Photons serve as robust quantum carriers with long coherence times, weak environmental coupling, and natural compatibility with integrated photonic circuits. Over the past decade, significant advances in silicon and silicon nitride photonics have enabled large‐scale programmable quantum circuits, multiphoton interference, and complex quantum state manipulation within chip‐scale systems [7, 8, 9, 10, 11, 12]. Two complementary paradigms currently dominate photonic quantum technologies. The first relies on probabilistic quantum light generation using nonlinear optical processes, such as spontaneous parametric down‐conversion and spontaneous four‐wave mixing, which underpin many demonstrations of multiphoton entanglement, boson sampling, and measurement‐based quantum computing [3, 7, 8, 11, 12]. The second paradigm, which has gained rapid momentum, is based on deterministic spin–photon qubits, where stationary matter qubits are interfaced with flying photonic qubits to enable on‐demand quantum state generation, quantum memory, and heralded remote entanglement [13, 14, 15, 16, 17]. In particular, silicon‐based spin–photon platforms, such as the T center, have recently demonstrated long‐lived electron and nuclear spin coherence, telecom‐wavelength optical interfaces, and multiqubit registers integrated within silicon photonic circuits, establishing silicon as a promising platform for distributed quantum technologies [17, 18, 19, 20, 21, 22, 23, 24].

Despite these advances, a critical bottleneck persists across both probabilistic and deterministic architectures: the lack of scalable high‐performance on‐chip single‐photon detectors operating at telecom wavelengths and near room temperature. Quantum photonic systems require detectors that simultaneously provide near‐unity photon detection efficiency, ultra‐low dark count rates, sub‐100 ps timing jitter, and multigigahertz electrical bandwidth to support quantum interference, fast spin readout, and high‐rate entanglement protocols [25, 26, 27]. However, existing detector technologies struggle to meet these requirements within a scalable and integrable platform. Conventional silicon single‐photon avalanche detectors (Si‐SPAD) are limited to visible wavelengths due to silicon's bandgap (∼1.1 eV) [28, 29, 30]. Superconducting nanowire single‐photon detectors (SNSPDs) offer exceptional efficiency and timing resolution but require cryogenic operation and complex on‐chip system‐level integration, which limits scalability for large photonic circuits [26, 31, 32, 33, 34]. InGaAs/InP SPADs operate in the telecom band but suffer from high dark count rates, afterpulsing, limited timing resolution, and poor compatibility with dense silicon photonic integration [25, 35, 36].

Germanium–silicon photodetectors offer a promising CMOS‐compatible platform for near‐infrared single‐photon detection due to their strong telecom‐band absorption and compatibility with silicon photonics. Unlike bulk Ge, which exhibits a direct bandgap near ∼0.8 eV, heteroepitaxial Ge‐on‐Si layers can exhibit slightly modified band structures due to tensile strain induced during epitaxial growth and thermal‐expansion mismatch with silicon. Previous studies have reported a direct‐gap value of approximately 0.784 eV for strained Ge‐on‐Si epilayers at room temperature [37, 38, 39, 40]. The tensile strain also modifies the near‐infrared absorption characteristics and absorption edge of Ge‐on‐Si heterostructures. In the telecom wavelength range (∼1.2–1.6 μm), strained Ge‐on‐Si heterostructures exhibit absorption coefficients ranging from approximately ∼6 × 10^3^ to ∼10^2^ cm^−1^ depending on wavelength, strain condition, and temperature‐dependent band‐structure effects [37]. These properties make Ge one of the leading absorption materials for monolithically integrated telecom‐band photodetectors and single‐photon avalanche detectors. At longer wavelengths (> 1.6 μm), the absorption behavior becomes increasingly sensitive to strain and temperature‐dependent indirect‐band effects. However, the performance of Ge/Si single‐photon avalanche detectors is often constrained by defects and interface trap states arising from lattice mismatch, which introduce excess dark current and timing jitter under Geiger‐mode operation [39]. Consequently, high‐performance Ge/Si SPADs require careful separation of the optical absorption and avalanche multiplication regions, such that photon absorption occurs in Ge while avalanche multiplication takes place in high‐quality silicon. This separation is projected to enable efficient carrier injection into the multiplication region while minimizing the impact of defects in the Ge layer.

Although significant progress has been made in Ge/Si avalanche photodiodes and SPADs, including waveguide‐integrated and lateral device geometries [38, 39, 40, 41, 42, 43, 44], key performance limitations remain. More recently, Ge‐on‐Si single‐photon avalanche detectors have been demonstrated in both vertical and lateral geometries, including waveguide‐coupled configurations [39, 40, 45]. Notably, recent studies have achieved room‐temperature Ge/Si SPAD operation through careful electrostatic‐field engineering to suppress dark current originating from defective Ge regions, representing an important milestone for Ge‐based single‐photon detection [41]. Nevertheless, the reported photon detection efficiencies remain modest, timing jitter often exceeds 100 ps, and device bandwidth is limited by parasitic capacitance and carrier transport. Furthermore, many existing demonstrations rely on free‐space illumination or weakly confined optical modes and are not optimized for nanophotonic waveguide integration required for scalable quantum photonic systems. For on‐chip quantum photonic platforms operating at telecom wavelengths, these limitations are particularly restrictive. High‐fidelity spin–photon interfaces, two‐photon interference, and heralded remote entanglement require detectors with high efficiency, low noise, and fast timing performance that exceed the capabilities of current Ge/Si SPAD implementations.

In this work, we address this challenge by proposing and validating a nanophotonic waveguide‐integrated Ge/Si single‐photon avalanche detector architecture through rigorous optical and electrical simulations. By combining strong optical confinement in nanophotonic waveguides, engineered photon absorption in germanium, and optimized avalanche multiplication in silicon, the proposed devices enable near‐unity internal quantum efficiency while maintaining low noise and high‐speed operation. As illustrated in Figure 1, these detectors are designed as an integral component of an all‐silicon quantum photonic platform, enabling scalable on‐chip single‐photon detection for probabilistic photonic sources, hybrid quantum photonic systems, and emerging silicon spin–photon qubit architectures.

**FIGURE 1:**
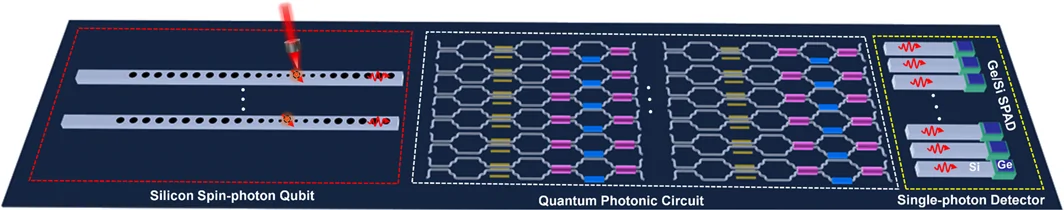
Conceptual schematic of an integrated silicon quantum photonic platform. On‐demand silicon spin–photon qubits generate single photons that are routed through a reconfigurable nanophotonic quantum circuit and detected on‐chip using waveguide‐integrated, high‐efficiency Ge/Si single‐photon avalanche detectors (SPADs). The monolithic integration of deterministic quantum light sources, low‐loss photonic circuitry, and near‐unity‐efficiency, low‐jitter single‐photon detectors enables scalable on‐chip quantum information processing, communication, and sensing.

## 2 Design of Nanophotonic Ge/Si Single‐Photon Avalanche Detectors

The proposed Ge/Si single‐photon avalanche detector is based on a nanophotonic separate‐absorption–multiplication (SAM) architecture that decouples optical absorption from avalanche multiplication while maintaining strong light–matter interaction, low noise, and high‐speed operation. This design addresses a fundamental limitation of conventional Ge/Si SPADs, where efficient absorption in Ge is often accompanied by increased dark current and excess noise due to carrier generation within high electrostatic field regions. By combining nanophotonic mode engineering with a carefully tailored electrostatic field profile, the present architecture resolves this trade‐off at the device level.

### 2.1 Optical Design and Nanophotonic Mode Engineering

The optical design of the detector was optimized using finite‐difference time‐domain (FDTD) simulations [46], which solve Maxwell's equations to model the propagation of guided optical modes from the input waveguide into the lateral Ge/Si SPAD integrated with nanophotonic structures. The wavelength‐dependent dielectric permittivity of the materials was modeled using a multi‐Lorentz oscillator formulation to accurately reproduce the refractive index dispersion and absorption characteristics of Ge [47]. Silicon absorption was assumed negligible within the telecom wavelength range considered in this work, whereas the absorption coefficient of Ge‐on‐Si was implemented using experimentally measured data [37]. Optical absorption within the Ge region was evaluated by integrating the electromagnetic power flux over the absorber volume, enabling direct quantification of the optical power dissipated in the Ge layer. A detailed formulation of the absorption modeling and internal quantum efficiency extraction, including optical field‐dependent power dissipation and volumetric integration, is provided in Supporting Information S1: Section SII. This approach allows accurate evaluation of photon absorption efficiency within the nanophotonic structure. Figure 2 illustrates the optical design strategy underlying the proposed detector architecture. A germanium absorption region is integrated on top of an intrinsic silicon layer that serves as the avalanche multiplication layer. To engineer the spatial distribution and confinement of the guided optical mode, photonic‐crystal nanostructures are patterned on both sides of the Ge/Si slab, forming a nanophotonic waveguide section that introduces a broadband photonic bandgap across the telecom wavelength range. This photonic bandgap suppresses lateral optical leakage and enhances optical confinement within the Ge absorber, enabling efficient photon absorption within a compact device footprint. The photonic bandgap design and its optimization in reciprocal space are analyzed in detail in Supporting Information S1: Section SI, where the band structure and confinement mechanism are explicitly quantified.

**FIGURE 2:**
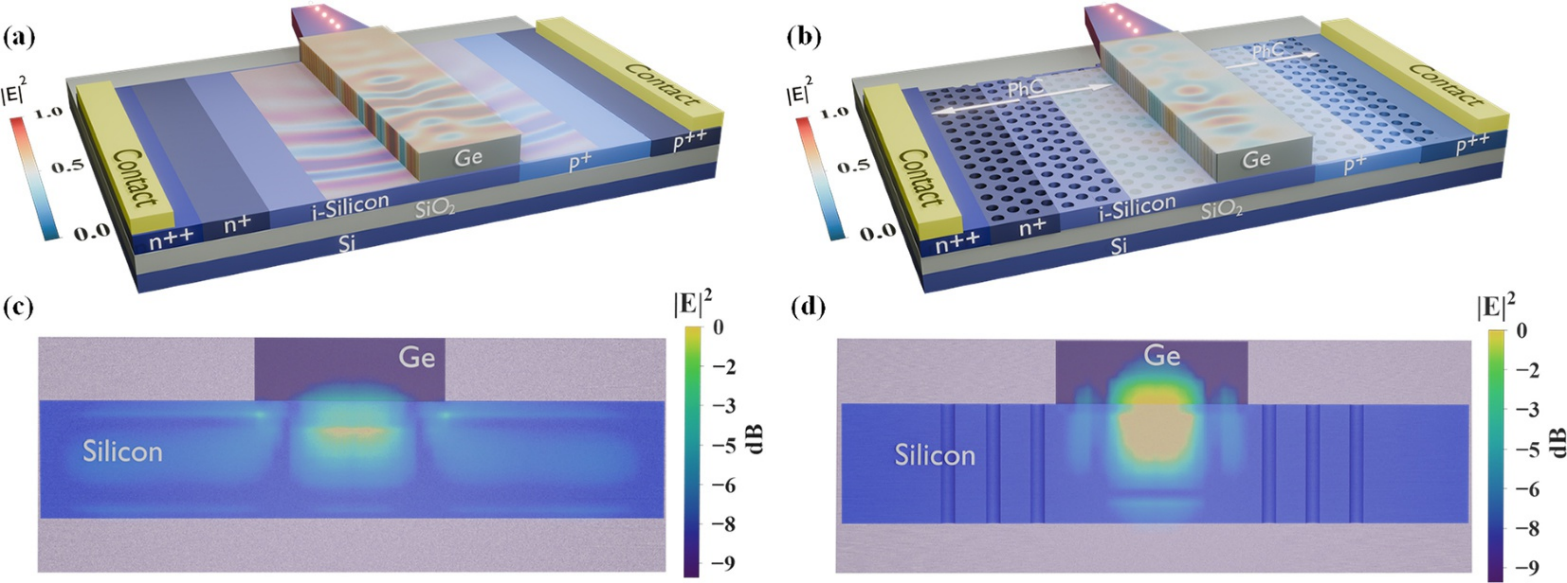
Nanophotonic Ge/Si separate‐absorption–multiplication (SAM) SPAD design and simulated optical field distributions. (a) Schematic of a waveguide‐integrated planar Ge/Si SAM SPAD coupled to an intrinsic silicon multiplication layer, with the simulated optical field distribution superimposed on the device geometry. (b) Corresponding nanophotonic design incorporating photonic‐crystal patterning on silicon to engineer the optical mode and enhance confinement within the Ge absorber, with the simulated optical field distribution superimposed on the device schematic. (c) Simulated cross‐sectional optical field intensity (|*E*|^2^) for the planar device, showing partial mode overlap with the Ge absorber and significant field penetration into the silicon multiplication region. (d) Simulated cross‐sectional optical field intensity (|*E*|^2^) for the nanophotonic device, demonstrating strong localization of the optical mode within the Ge absorber and suppressed field overlap with the high‐field silicon avalanche region. The nanophotonic architecture enables compact and efficient photon absorption in Ge while maintaining spatial separation from the high‐electrostatic‐field avalanche region confined to intrinsic silicon. This nanophotonic SAM design suppresses defect‐ and field‐assisted noise mechanisms while preserving fast carrier transport and low junction capacitance.

In Figure 2a–d, the FDTD simulated optical field distributions are superimposed on the corresponding device schematics to directly correlate modal confinement with the physical Ge/Si geometry and nanophotonic patterning. In the planar reference geometry shown in Figure 2a, the optical mode is only weakly confined to the Ge region. The corresponding simulated cross‐sectional optical field distribution in Figure 2c confirms that a significant fraction of the optical field penetrates into the silicon multiplication region. Although this geometry enables waveguide‐integrated absorption, it increases the probability of carrier generation in regions subject to strong electrostatic fields, which is undesirable for low‐noise Geiger‐mode operation. The nanophotonic design shown in Figure 2b introduces periodic patterning with hexagonal lattice on both sides of the Ge/Si slab to reshape the guided optical mode. The photonic‐crystal geometry suppresses radiation loss and redistributes the modal optical field, resulting in strong localization within the Ge absorber while maintaining only evanescent coupling to the underlying silicon. The superimposed simulated optical fields in Figure 2b visually demonstrate this enhanced confinement relative to the planar case. This behavior is further confirmed by the simulated cross‐sectional optical field profile in Figure 2d, where the optical field is tightly confined to a compact Ge volume with strongly suppressed overlap in the silicon multiplication region. Importantly, this enhanced confinement is achieved without increasing the Ge thickness or relying on narrowband resonant effects, enabling efficient absorption over short interaction lengths while preserving broadband operation across the telecom band. As a result, the nanophotonic optimized architecture enables efficient photon absorption while enforcing the spatial separation required for low‐noise avalanche multiplication.

### 2.2 Electrical Design and Separate‐Absorption–Multiplication Operation

The nanophotonic and electrical designs are co‐engineered to establish a true SAM architecture, enabling strong optical absorption within the Ge region while spatially confining the high‐electrostatic‐field avalanche multiplication process to silicon. In this configuration, the Ge absorption layer is positioned above an intrinsic silicon region that serves as the avalanche multiplication layer under reverse bias. Lateral *n* ^+^/*p* ^+^ and *n* ^++^/*p* ^++^ doped regions are incorporated to establish a strong electrostatic field across the intrinsic silicon while enabling efficient carrier injection and collection through metal contacts. Under Geiger‐mode operation, the electrostatic field within the intrinsic silicon is engineered to exceed the threshold required for avalanche multiplication, whereas the Ge absorption region remains outside the peak electrostatic field region. To maintain high‐speed operation despite the reduced electrostatic field in Ge, the absorber thickness is intentionally minimized (∼140 nm), and the nanophotonic geometry localizes carrier generation near the Ge/Si transfer interface, thereby reducing diffusion‐limited carrier transit delays. This spatial separation suppresses tunneling, trap‐assisted carrier generation, and field‐enhanced leakage currents originating from defects in Ge. At the same time, photogenerated carriers created in the Ge absorber are efficiently transferred into the silicon multiplication region, enabling high internal gain while maintaining low noise. The separation of optical absorption and high‐electrostatic field avalanche multiplication is a defining characteristic of SAM architecture and is further reinforced by the nanophotonic optical design. By minimizing optical field overlap with the high‐electrostatic field region, photon absorption occurs predominantly within the Ge absorber rather than within the avalanche region, thereby reducing noise processes that typically limit room‐temperature SPAD operation.

#### 2.2.1 Electrostatic Field Modeling

The electrostatic potential distribution within the device was obtained by solving the Poisson equation $\nabla^{2}\phi =\frac{q}{\varepsilon \varepsilon_{0}}\left(n+N-p-P\right)$, where ϕ is the electrostatic potential, n and p are the electron and hole concentrations, and N and P represent the donor and acceptor doping densities. The field is obtained from the gradient of the potential, E=−∇ϕ. The simulations incorporate the full device geometry including the photonic‐crystal nanostructures, doping profiles, and applied reverse bias. Boundary conditions are defined at the metal contacts whereas zero normal potential gradient (∇ϕ=0) is applied at insulating boundaries. In the simulations, the highly doped regions are defined as $n^{++}=p^{++}=10^{19}$ cm^−3^ and $n^{+}=p^{+}=10^{17}$ cm^−3^ (Supporting Information S1: Figure S3a), which establish the lateral electrostatic field profile required for avalanche multiplication in the intrinsic silicon region.

#### 2.2.2 Carrier Dynamics and Avalanche Modeling

Carrier transport and avalanche multiplication dynamics were evaluated using Monte Carlo simulations [48]. Photogenerated electron–hole pairs were generated with probabilities proportional to the optical absorption profile obtained from the FDTD simulations. Carriers undergo stochastic transport events including ionization, recombination, and inelastic scattering. The free‐flight distance between scattering events is determined using $r=r_{0}\;\ln\left(\text{rand}\right)$, where $r_{0}$ represents the mean free path associated with the scattering process and rand is a uniformly distributed random variable. Ionization events occur primarily in the silicon multiplication region due to the high electrostatic field present in this layer. Impact‐ionization coefficients for silicon were taken from established experimental measurements [49]. The avalanche current pulse generated at the contacts is recorded for each simulated event. From these transient current pulses, the avalanche gain, timing jitter, and electrical bandwidth are extracted. The detector bandwidth is estimated from the 3‐dB cutoff frequency obtained through fast Fourier transform (FFT) analysis of the current pulse response.

#### 2.2.3 Dark Current and Dark Count Rate Modeling

The present work focuses on physics‐guided comparative device design and electro‐optical optimization rather than a fully calibrated predictive SPAD compact model. Dark current was calculated by considering carrier‐generation mechanisms in the absence of optical excitation. The carrier generation rate (CGR) includes contributions from thermal generation, diffusion processes, band‐to‐band tunneling, surface generation, and afterpulsing effects [49]. The bulk silicon region is assumed to have a low impurity concentration of $N_{t}=10^{10}-10^{11}$ cm^−3^, whereas the device surfaces are assumed to be well passivated with interface trap densities of $D_{it}=10^{9}-10^{10}$ cm^−2^ eV^−1^ [37, 50, 51].

The surface dark current is proportional to the surface recombination velocity $s_{0}=\sigma v_{\text{th}}kT,$ where σ is the carrier capture cross section, $v_{\text{th}}$ is the carrier thermal velocity, k is the Boltzmann constant, and T is the temperature. The dark count rate (DCR) is then expressed as $\text{DCR}=\text{CG}R_{\text{total}}\;P_{\text{th}}$, where $P_{\text{th}}$ is the avalanche triggering probability. This probability depends on the breakdown voltage $V_{\text{brk}\;}$ and the excess bias voltage $V_{\text{EX}}=V-V_{\text{brk}}$ and is approximated as $P_{\text{th}}=1-\exp(-\frac{V_{\text{EX}}}{\eta_{T}V_{\text{brk}}})$, where $\eta_{T}$ is an empirical parameter with $\eta_{T}\approx 0.131$ [49, 52]. Additional details of the Monte Carlo carrier transport model and avalanche gain characteristics are provided in Supporting Information S1: Section SIII. The present dark‐count‐rate analysis is based on a carrier‐generation‐rate (CGR) framework combined with electrostatic field simulations, providing a first‐order comparative estimation of thermally generated carrier contributions within the Ge/Si SAM architecture. The model captures the influence of electrostatic‐field distribution, absorber geometry, and nanophotonic confinement on the relative dark‐current behavior. Additional mechanisms can contribute to the experimental SPAD DCR, including trap‐assisted tunneling, surface‐state generation, afterpulsing, defect‐assisted avalanche retriggering, and process‐dependent interface traps. These effects depend strongly on fabrication quality and experimentally calibrated defect parameters and are therefore not explicitly included in the present simulations. Consequently, the reported DCR‐related results should be interpreted as comparative device‐level electrostatic estimates rather than absolute experimental DCR predictions.

#### 2.2.4 Electrostatic Field Engineering With Photonic‐Crystal Nanostructures

The photonic‐crystal nanostructures used for optical confinement also influence the local electrostatic‐field distribution. Due to the refractive‐index discontinuity between silicon and the air hole, the electrostatic field satisfies the boundary condition $\varepsilon_{\text{Si}}E_{n,\text{Si}}=E_{n,\text{air}}$, where $E_{n\;}$ represents the normal electrostatic field component at the Si/air interface. This discontinuity leads to localized distortion of the electrostatic field near the nanoholes, resulting in a narrower high‐field region within the silicon multiplication layer. This localized electrostatic field enhancement increases the probability of impact ionization within the multiplication region while maintaining a compact avalanche zone. Compared with conventional doping‐defined high‐electrostatic field regions, the photonic‐crystal geometry further confines the avalanche region, enabling reduced avalanche buildup time and improved timing jitter. The higher local electrostatic field also reduces the voltage required to reach Geiger‐mode operation. Careful alignment of the doping profile and photonic‐crystal geometry is required to ensure that the peak electrostatic field occurs near the final photonic‐crystal row. In this configuration, photogenerated carriers can efficiently enter the multiplication region without being scattered by the nanostructures, thereby minimizing additional surface‐generated dark current.

Together, the engineered electrostatic‐field distribution and nanophotonic waveguide architecture can enable efficient carrier injection into a confined silicon avalanche region while suppressing defect‐induced noise from the Ge absorption layer. The SAM configuration ensures that photon absorption occurs primarily within the Ge region, whereas avalanche multiplication is confined to high‐quality intrinsic silicon, thereby minimizing dark current and excess noise mechanisms that typically limit Ge/Si detectors. At the same time, the localized electrostatic‐field enhancement induced by the photonic‐crystal geometry reduces avalanche buildup time and improves carrier multiplication efficiency. As a result, the combined optical and electrical design provides a pathway toward waveguide‐integrated Ge/Si SPADs capable of achieving high photon detection efficiency, low timing jitter, and multigigahertz bandwidth required for scalable quantum photonic systems.

### 2.3 Design Realism and Fabrication Compatibility

The proposed nanophotonic Ge/Si SAM SPAD architecture is intentionally developed to be compatible with established CMOS fabrication workflows. Devices are designed on silicon‐on‐insulator substrates with a 220‐nm silicon device layer, consistent with standard silicon photonics platforms. In the present design, the Ge absorber thickness is optimized to 140 nm, balancing strong optical absorption with efficient carrier transfer into the silicon multiplication region. Key nanophotonic design parameters, including photonic‐crystal periodicity and hole diameter, are treated as adjustable variables and optimized to achieve broadband optical confinement in the telecom band. These parameters are further explored as sweeping variables in the following section to elucidate their influence on detector performance. The photonic‐crystal holes are implemented exclusively within the surrounding silicon regions rather than the Ge absorber itself in order to preserve Ge material quality and suppress defect‐assisted leakage pathways associated with etched Ge sidewalls.

To preserve Ge material quality during the fabrication process, all silicon doping required to form the SAM junction can be defined and electrically activated prior to Ge integration using selective ion implantation [39]. Germanium can then be integrated deterministically by selective‐area epitaxy through patterned dielectric windows, enabling precise control over the absorber placement and thickness [53]. Subsequent electron‐beam lithography and anisotropic etching can be performed to define the nanophotonic absorber geometry and photonic‐crystal patterning. Conformal dielectric passivation (e.g., SiO_2_ or AlO_2_) can be employed to suppress Ge surface recombination and leakage currents in Ge, thereby stabilizing device operation, followed by standard metallization to form complete electrical contacts [39, 41]. Importantly, the process flow avoids high‐temperature steps after Ge integration, confines high electrostatic fields to intrinsic silicon, and preserves the integrity of the Ge absorber. As such, the proposed design represents a realistic and manufacturable pathway toward waveguide‐integrated Ge/Si SPADs compatible with CMOS processing and scalable silicon photonics.

## 3 Results and Discussion

### 3.1 Broadband Near‐Unity Internal Quantum Efficiency Enabled by Nanophotonic Mode Confinement

Figure 3 quantifies the optical absorption performance of the proposed Ge/Si SAM SPAD architecture and directly highlights the advantage of nanophotonic mode engineering over a planar Ge/Si geometry. In Figure 3a, the simulated internal quantum efficiency (IQE) is plotted as a function of wavelength for two Ge absorber lengths (4 and 16 μm) comparing a planar (“flat”) waveguide to the nanophotonic (photonic‐crystal, PhC) design. The IQE is extracted from the simulated absorption profile (see Supporting Information S1: Section SII). For the nanophotonic device, IQE approaches unity across a broad wavelength window spanning the telecom band, whereas the planar device exhibits substantially lower IQE, particularly for the compact 4 μm absorber length. This behavior is consistent with the optical field profiles shown previously in Figure 2: the planar structure supports only partial overlap with Ge and allows significant mode leakage into surrounding regions, whereas the photonic‐crystal patterning reshapes the guided mode and enhances confinement within the Ge absorber. It should be noted that the planar device serves as a reference structure rather than a separately optimized Ge photodetector. Previous Ge waveguide photodetectors have achieved higher absorption efficiencies through larger Ge volumes, longer interaction lengths, and optimized optical coupling geometries [43, 54, 55]. In contrast, the compact Ge absorber employed here is intentionally chosen to reduce carrier transit time, junction capacitance, and Ge‐related leakage mechanisms that contribute to dark current and dark‐count rate. The proposed nanophotonic architecture mitigates the resulting absorption–speed–noise trade‐off by enhancing optical confinement without increasing the absorber volume.

**FIGURE 3:**
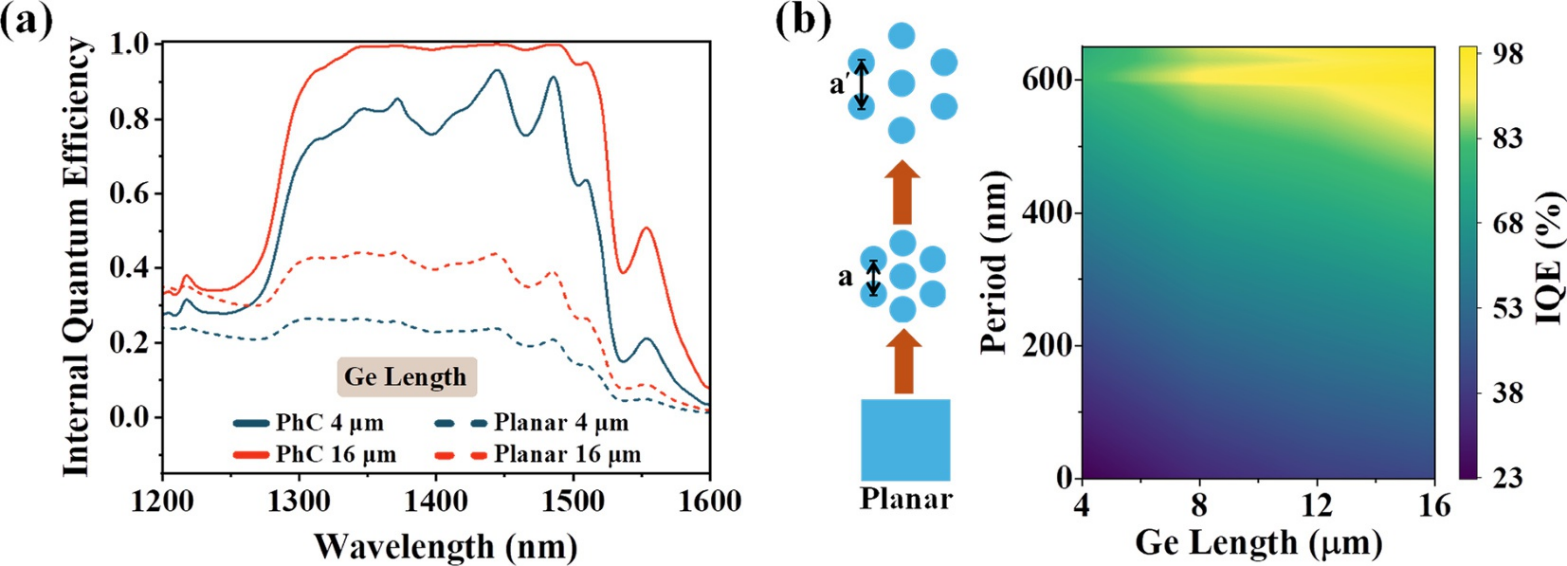
Simulated internal quantum efficiency (IQE) of nanophotonic Ge/Si SAM SPADs. (a) Wavelength‐dependent internal quantum efficiency (IQE) for planar and nanophotonic (photonic‐crystal, PhC) Ge/Si SAM SPADs with Ge absorber lengths of 4 and 16 μm. The nanophotonic design exhibits near‐unity IQE across a broad telecom wavelength range, significantly outperforming the planar geometry, particularly for short absorber lengths. (b) Simulated IQE as a function of Ge absorber length and photonic‐crystal periodicity near 1450 nm wavelength, illustrating the transition from planar to nanophotonic regimes. Increasing nanophotonic confinement and absorber length enables high IQE (> 95%) over compact device footprints, highlighting the role of nanophotonic mode engineering in enhancing absorption efficiency while preserving scalability.

Two points are particularly important from Figure 3a. First, the nanophotonic enhancement is most pronounced for short absorber lengths. This is technologically significant because compact Ge lengths reduce capacitance, enable higher bandwidth, and mitigate carrier transit‐time dispersion, parameters that directly influence timing jitter and speed. Second, the high IQE is maintained over a wide spectral range rather than relying on a narrow resonance. A comprehensive wavelength‐dependent comparison across multiple absorber lengths is also provided in Supporting Information S1: Figure S2.

This broadband behavior is consistent with a photonic‐bandgap–assisted confinement mechanism rather than a high‐Q cavity effect, making the design robust to fabrication tolerances and compatible with multiwavelength quantum photonic circuits. Figure 3b provides a more global view of the design space by mapping IQE as a function of Ge length and photonic‐crystal periodicity. The monotonic improvement of IQE with Ge length reflects the expected increase in absorption probability with interaction length; however, the periodicity‐dependent trend reveals the role of nanophotonic confinement in achieving high efficiency without requiring long absorbers. As the periodicity is tuned deeper into the nanophotonic regime, the optical mode becomes increasingly localized within Ge, enabling high IQE (> 90%–95%) over compact footprints. This design degree of freedom is critical for integrated quantum photonics because it allows absorption to be optimized independently from the electrical multiplication region and independently from circuit‐level constraints such as waveguide routing density and device placement. Collectively, Figure 3 establishes that the nanophotonic Ge/Si SAM architecture can deliver near‐unity quantum efficiency over the telecom band while maintaining compact absorber lengths. This combination is a prerequisite for scalable on‐chip single‐photon detection in quantum photonic processors and network nodes, where detector footprint, power, and bandwidth are tightly constrained.

### 3.2 Electrostatic Field Engineering for SAM Operation and Low‐Noise Geiger‐Mode Detection

Although high quantum efficiency is necessary, it is not sufficient for room‐temperature single‐photon detection; the detector must also suppress noise mechanisms that scale strongly with electrostatic field and material defect density. Figure 4 addresses this requirement by analyzing the electrostatic‐field distribution and bias dependence of the proposed nanophotonic Ge/Si SAM SPAD. Figure 4a compares simulated electrostatic field magnitude distributions for the nanophotonic and planar Ge/Si geometries under breakdown voltages of 18 and 28 V, respectively. In the nanophotonic design, the electrostatic field is very narrow and strongly confined to the intrinsic silicon multiplication region, whereas the Ge absorption region remains outside the peak‐field zone. This separation‐of‐absorption and multiplication is central to suppressing defect‐assisted and electrostatic field‐assisted carrier generation processes in Ge (e.g., trap‐assisted tunneling and field‐enhanced leakage), which are widely recognized as dominant contributors to dark current and dark count rate in Ge/Si devices operating near/at room temperature. At the same time, the absorber remains efficiently coupled optically (Figure 3) and electrically through carrier transfer into the silicon multiplication layer, enabling high internal gain without placing Ge in the high‐electrostatic field avalanche region. In contrast, the planar geometry exhibits weaker electrostatic field localization and reduced control over the field distribution. This lack of field confinement makes it more difficult to simultaneously achieve strong avalanche multiplication and low noise, because unwanted high field regions can extend toward the absorber or interfaces where defect densities and leakage pathways are higher. Therefore, the combination of Figures 3 and 4 highlights a key advantage of the proposed architecture: nanophotonic confinement increases absorption efficiency in Ge, whereas electrostatic field engineering confines avalanche multiplication to intrinsic silicon, enabling low‐noise near‐unity‐efficiency single‐photon detection at room temperature.

**FIGURE 4:**
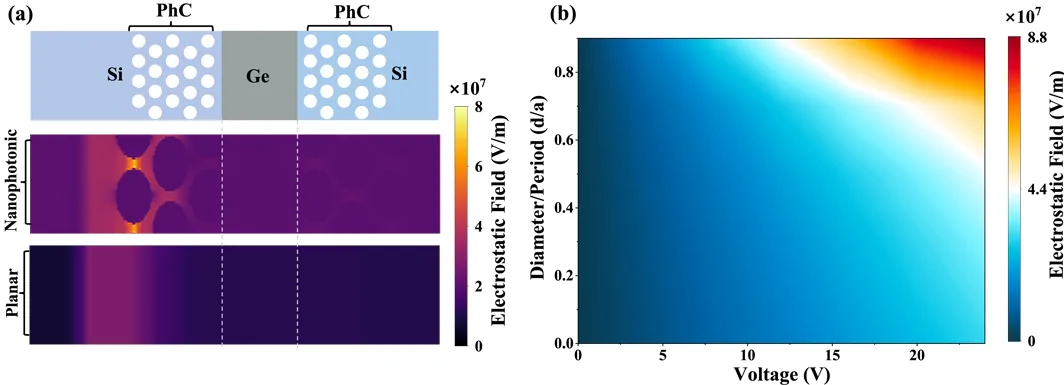
Electrostatic‐field distribution and bias dependence in nanophotonic Ge/Si SAM SPADs. (a) Top‐view schematic and simulated electrostatic‐field magnitude distributions for nanophotonic (period 610 nm and hole diameter 530 nm) and planar Ge/Si SAM SPAD geometries under breakdown voltage of 18 and 28 V, respectively. The photonic‐crystal holes are patterned exclusively within the adjacent silicon nanophotonic regions surrounding the Ge absorber, whereas the central Ge absorption region remains unetched and physically isolated from the nanophotonic holes. In the nanophotonic design, the high electrostatic field is strongly confined to the intrinsic silicon multiplication region, whereas the Ge absorption region remains outside the peak‐field zone. This field separation suppresses defect‐ and field‐assisted noise processes in Ge while preserving efficient photon absorption and near‐unity internal quantum efficiency under idealized carrier‐collection conditions. In contrast, the planar geometry exhibits weaker field localization and reduced control over the electrostatic‐field distribution. (b) Simulated peak electrostatic field as a function of applied reverse bias and photonic‐crystal periodicity, illustrating systematic field enhancement and tunability enabled by the nanophotonic architecture. The nanophotonic design supports strong and controllable avalanche fields in silicon while maintaining separation from the Ge absorber, a key requirement for low‐noise Geiger‐mode operation.

Although the proposed nanophotonic architecture incorporates photonic‐crystal hole patterning, the holes are implemented primarily within the surrounding silicon regions rather than directly within the Ge absorber. Consequently, the Ge absorption layer remains physically unetched, minimizing Ge‐related surface defects and defect‐assisted leakage pathways. In addition, the nanophotonic patterning reduces the effective junction area and capacitance, which supports fast temporal response and low‐jitter avalanche operation. The electrostatic field is engineered such that the peak field remains localized within the buried silicon multiplication region, while comparatively weaker electrostatic fields are maintained near exposed nanophotonic surfaces and within the Ge absorber. This electrostatic‐field separation suppresses surface‐assisted carrier injection, trap‐assisted tunneling, and Ge‐related leakage mechanisms that contribute to elevated dark‐count rate. The simulations further assume effective surface passivation conditions to reduce surface trap‐assisted generation and recombination effects. The simulated breakdown voltage (*V*_BD_) is ∼18 V for the nanophotonic device and ∼28 V for the planar structure. The corresponding breakdown behavior and avalanche gain scaling with bias are further analyzed in Supporting Information S1: Figure S3.

Figure 4b further shows the tunability of the peak electrostatic field as a function of applied reverse bias and photonic‐crystal geometry. The nearly monotonic increase of the peak electrostatic field with reverse bias is expected; however, the dependence on hole diameter‐to‐period ratio (d/p) indicates that the nanophotonic patterning also influences the effective electrostatic‐field distribution through geometry‐dependent field crowding and depletion shaping. This provides an important co‐design parameter, where d/p can be optimized not only for optical confinement (Figure 3) but also to support strong and controllable avalanche multiplication fields in silicon while maintaining electrostatic separation from the Ge absorber. Such electro‐optic co‐optimization is particularly important for achieving Geiger‐mode operation with high gain while suppressing thermally activated leakage pathways at room temperature.

Therefore, Figures 3 and 4 establish that the nanophotonic Ge/Si SAM SPAD simultaneously satisfies two stringent and often competing requirements: (i) broadband optical absorption in a compact Ge absorber enabled by nanophotonic mode engineering, and (ii) high‐electrostatic field confinement to intrinsic silicon enabled by SAM electrostatics. These results provide the foundation for the time‐domain and noise performance trends analyzed next, where timing response, bandwidth, and dark‐current/dark‐count scaling is evaluated as a function of key geometrical parameters.

### 3.3 Temporal Response, Bandwidth, and Geometry‐Dependent Optimization

Beyond efficient optical absorption and electrostatic‐field confinement, high‐speed single‐photon detection requires rapid carrier transport, low junction capacitance, and minimal temporal dispersion. In the proposed SAM architecture, the electric field within the Ge absorption region is intentionally maintained at a moderate level to suppress defect‐assisted dark current and tunneling‐related noise. Nevertheless, residual electric‐field components together with built‐in electrostatic gradients near the Ge/Si interface continue to assist carrier transfer toward the silicon multiplication region. In addition, the Ge absorber is intentionally kept thin (∼140–150 nm), whereas the nanophotonic architecture localizes the optical mode near the Ge/Si interface, thereby reducing the effective carrier transport distance. Under these conditions, carrier transport occurs through the combined contribution of drift and diffusion with subpicosecond‐to‐picosecond‐scale transit dynamics. Prior studies on thin Ge‐on‐Si photodetectors have demonstrated ultrafast carrier transport and bandwidths extending from tens to hundreds of gigahertz under moderate electric‐field conditions [56]. As a result, the proposed nanophotonic Ge/Si SAM architecture can preserve fast temporal response while maintaining reduced electric‐field intensity within the Ge absorber for low‐noise operation. Figure 5 summarizes the simulated time‐domain and frequency‐domain response of the proposed device together with the influence of key geometrical parameters, including Ge absorber length, intrinsic silicon width, and photonic‐crystal periodicity, on timing response, bandwidth, and dark‐current behavior. The interplay among avalanche gain, electrostatic‐field confinement, and breakdown characteristics is further discussed in Supporting Information S1.

**FIGURE 5:**
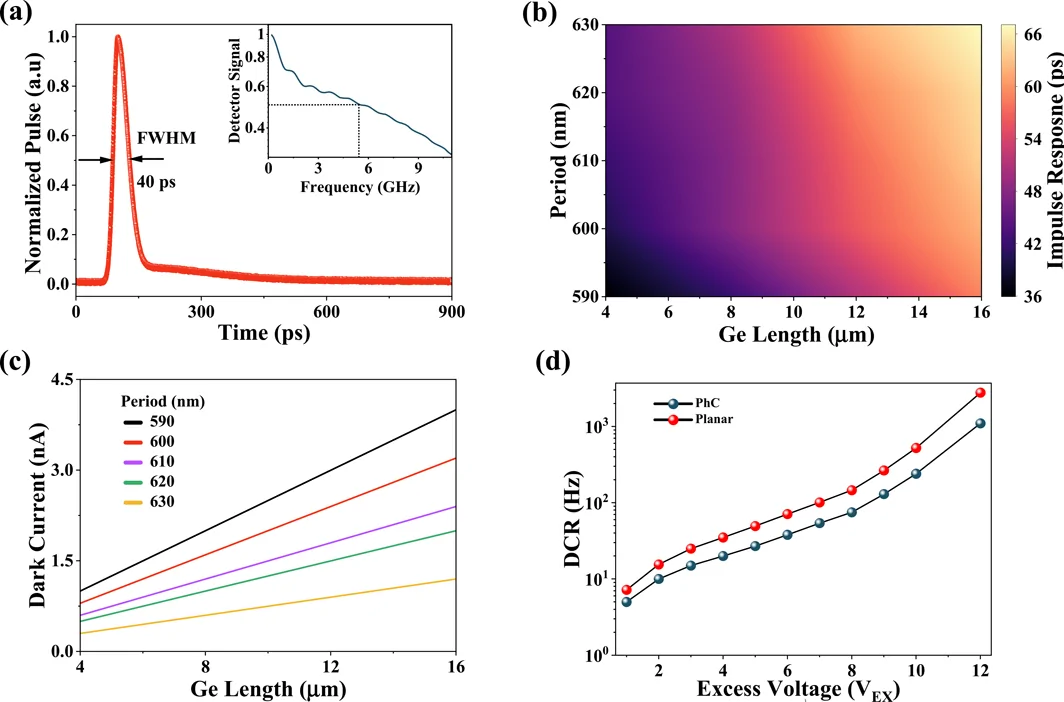
Temporal response, bandwidth, and geometry‐dependent performance of nanophotonic Ge/Si SAM SPADs. (a) Simulated intrinsic impulse response of the nanophotonic Ge/Si SAM SPAD, illustrating fast carrier transport and avalanche‐response dynamics within the separate‐absorption–multiplication architecture at reverse biasing of 18 V. The extracted full‐width‐at‐half‐maximum (FWHM) temporal response is below 40 ps under the simulated operating conditions. The inset shows the corresponding small‐signal frequency response showing an intrinsic 3‐dB electro‐optical bandwidth exceeding 5 GHz, enabled by reduced carrier transit time, low junction capacitance, and efficient electrostatic‐field confinement within the silicon multiplication region. (b) Simulated impulse response as a function of Ge absorption length and photonic‐crystal period, illustrating the geometry‐dependent trade‐off between optical absorption and carrier transit time. The map identifies the parameter regime that minimizes temporal response while maintaining strong optical confinement in the Ge absorber. (c) Simulated dark current as a function of Ge absorption length at 18 V reverse bias for different photonic‐crystal periods. The results indicate that optimized nanophotonic geometries can maintain low leakage current while preserving efficient carrier injection into the silicon multiplication region. (d) Simulated dark‐count‐rate (DCR) as a function of excess voltage (*V*_EX_) for the nanophotonic and planar Ge/Si SAM‐SPAD structures. The nanophotonic architecture demonstrates lower DCR than the planar reference device over the investigated excess‐bias range.

Figure 5a shows the simulated temporal impulse response of the nanophotonic Ge/Si SAM SPAD under Geiger‐mode operation. The response exhibits a sharp leading edge followed by a rapidly decaying tail, indicating efficient carrier transfer from the Ge absorption region into the silicon multiplication layer and rapid avalanche buildup. The fast temporal response is enabled by several aspects of the proposed nanophotonic Ge/Si SAM architecture. Although the electrostatic field within the Ge absorption region is intentionally reduced to suppress field‐assisted dark‐current generation, the Ge layer itself is designed to be very thin (∼140 nm), substantially reducing the carrier transport distance prior to injection into the silicon multiplication region. In addition, the nanophotonic structure spatially localizes the optical mode near the Ge/Si carrier‐transfer interface, resulting in carrier generation in close proximity to the multiplication region and thereby minimizing diffusion‐limited delay. Similar high‐speed carrier‐transport strategies have previously been demonstrated in Ge/Si separate‐absorption–multiplication photodetectors and avalanche photodiodes, where compact absorption regions and short carrier‐transfer distances enabled bandwidths exceeding tens of gigahertz [56]. In addition, ultrafast nanophotonic thin‐film silicon photodetectors have demonstrated temporal responses as low as ∼31 ps FWHM through reduced carrier transit distance and low‐capacitance device geometries [57, 58]. Hence, the reduced device capacitance associated with the compact nanophotonic geometry further contributes to the sub‐40 ps temporal response and multigigahertz bandwidth observed in the simulations.

From the temporal profile, a full width at half maximum (FWHM) of approximately 40 ps is extracted, demonstrating the capability of the proposed architecture to support low timing jitter. The fast temporal response arises from the SAM configuration, which confines avalanche multiplication to the intrinsic silicon region while maintaining efficient carrier injection from the Ge absorber. This architecture reduces carrier diffusion and minimizes transit delays that typically broaden the timing distribution in conventional Ge/Si photodetectors. The corresponding frequency‐domain response is shown in the inset of Figure 5a. The Fourier transform of the temporal impulse response reveals a 3 dB bandwidth of approximately 5–6 GHz, consistent with the short temporal response observed in Figure 5a. This multigigahertz bandwidth results from the compact device geometry, reduced junction capacitance, and efficient carrier transport within the silicon multiplication region. Importantly, such bandwidth is achieved without aggressive scaling of the absorber thickness or operation seat cryogenic temperatures, highlighting the suitability of the nanophotonic Ge/Si SAM architecture for high‐speed single‐photon detection at or near room temperature. The sub‐40 ps pulse response and higher bandwidth (5–6 GHz) represent the intrinsic electro‐optical response capability of the proposed Ge/Si nanophotonic SAM design under the simulated operating conditions. However, in a practical SPAD design the achievable count rate can be limited by afterpulsing, trap‐assisted carrier release, dead time, and dark‐count‐rate. The Monte Carlo transport model used in this work includes the carrier drift, diffusion, recombination, multiplication, thermal generation, band‐to‐band tunneling, surface‐generation effects, and afterpulsing. These mechanisms primarily contribute to the temporal pulse broadening and extended decay tails. Additionally, nanophotonic patterning can introduce surface defects and trap states that may increase the dark count rate and afterpulsing probability if surface passivation is insufficient. In the present design, photonic crystal holes are designed on surrounding silicon region, whereas the Ge region thickness is kept thin intentionally and operated under low electrostatic field to suppress defect‐assisted tunneling. The simulations further assume effective surface passivation conditions to minimize surface‐generation contributions. Experimentally realized SPAD devices may benefit from the quenching electronics and gated voltage to further suppress the afterpulsing effects.

Figure 5b,c examine the influence of geometrical design parameters on device performance. Figure 5b presents the simulated impulse response as a function of Ge absorption length and photonic‐crystal period. Increasing the Ge length improves photon absorption probability but simultaneously increases carrier transit distance, leading to a gradual increase in temporal response. Similarly, the photonic‐crystal period affects optical confinement and the spatial distribution of the electrostatic field. The parameter map identifies a region in which the temporal response remains below approximately 40–45 ps while maintaining efficient optical confinement within the Ge absorber. Figure 5c shows the estimated dark current as a function of Ge absorption length for several photonic‐crystal periods. As expected, increasing the absorber length increases the probability of thermally generated carriers and therefore leads to higher dark current. However, larger photonic‐crystal periods reduce the overlap between the optical mode and high‐electrostatic field regions, thereby mitigating leakage currents while preserving efficient carrier injection into the silicon multiplication layer. The simulated reverse‐bias dark‐current characteristics of the proposed Ge/Si SAM–SPAD structure exhibit low dark current at moderate reverse‐bias voltages, followed by a rapid increase as the device approaches the high‐field avalanche regime near ∼30 V (Supporting Information S1: Figure S4). This behavior is consistent with enhanced field‐assisted carrier generation and avalanche multiplication under high electrostatic‐field conditions. Nevertheless, owing to the SAM architecture, the highest electrostatic field remains confined within the silicon multiplication region whereas the Ge absorber experiences a comparatively lower field. This electrostatic‐field separation suppresses defect‐assisted carrier generation in Ge and helps maintain low projected dark‐count rates over a broad operating range as further illustrated by the simulated DCR characteristics in Figure 5d.

Figure 5d shows the simulated DCR as a function of excess voltage for both the nanophotonic and planar Ge/Si SPAD structures. As expected, DCR increases with increasing excess bias due to the enhanced avalanche‐triggering probability and field‐assisted carrier‐generation mechanisms under higher electric‐field conditions. Notably, the nanophotonic device consistently exhibits a lower DCR than the planar reference structures across the entire investigated excess‐bias range while simultaneously providing significantly enhanced optical absorption and IQE. The result demonstrates that the IQE enhancement achieved by the nanophotonic design does not occur at the expense of increased detector noise. The improvement originates from the SAM architecture, which confines the highest electrostatic field within the silicon multiplication region while maintaining comparatively moderate electric‐field conditions in the Ge absorber. Consequently, dominant Ge‐related dark‐current mechanisms, including defect‐assisted carrier generation, trap‐assisted tunneling, and band‐to‐band tunneling, are suppressed. Furthermore, the nanophotonic design enhances optical absorption through photonic‐crystal‐assisted mode confinement rather than by increasing the Ge absorption volume, thereby avoiding a corresponding increase in dark‐current generation. These results highlight the importance of co‐optimizing nanophotonic optical confinement and electrostatic‐field engineering. Hence, the results in Figure 5 demonstrate that the proposed nanophotonic Ge/Si SAM SPAD architecture is projected to achieve sub‐40 ps temporal response and multigigahertz bandwidth while providing geometric design flexibility to optimize speed and noise characteristics. Furthermore, the detailed optical, electrical, and avalanche modeling supporting these results is provided in the Supporting Information S1. Combined with the strong optical confinement and efficient photon absorption discussed in the previous sections, these findings establish the nanophotonic Ge/Si SAM detector as a promising platform for high‐speed, low‐noise, room‐temperature single‐photon detection in integrated quantum photonic systems.

## 4 Summary

This work presents a nanophotonic waveguide‐integrated Ge/Si single‐photon avalanche detector based on a SAM architecture for high‐performance room‐temperature single‐photon detection in integrated quantum photonic platforms. By combining photonic‐crystal–enabled optical mode engineering with tailored electrostatic‐field confinement, the proposed design spatially separates efficient photon absorption in germanium from avalanche multiplication in intrinsic silicon. This approach addresses key limitations of conventional Ge/Si SPADs by simultaneously improving photon absorption efficiency, reducing defect‐related noise, and enabling fast carrier multiplication. Nanophotonic confinement localizes the guided optical mode within a compact Ge absorption region, which can enable near‐unity photon detection efficiency across the telecom wavelength range while maintaining device dimensions compatible with dense photonic integration. At the same time, the electrostatic design confines high electrostatic fields to the silicon multiplication layer, suppressing field‐assisted leakage currents and enabling stable Geiger‐mode operation at room temperature. Device‐level electro‐optical simulations further indicate a sub‐40 ps temporal impulse response and multigigahertz electrical bandwidth exceeding 5 GHz, demonstrating the potential for high‐speed single‐photon detection. A systematic exploration of key geometrical parameters, including Ge absorber length and photonic‐crystal periodicity, reveals a flexible design space that can enable co‐optimization of absorption efficiency, timing response, and dark current. Importantly, the proposed device architecture is compatible with CMOS fabrication on silicon‐on‐insulator platforms, providing a realistic pathway toward large‐scale integration. Taken together, these results establish nanophotonic Ge/Si SAM SPADs as a promising detector platform for compact, high‐efficiency, and high‐speed single‐photon detection, with direct relevance to integrated quantum computing, quantum communication, and on‐chip quantum sensing systems. More broadly, the integration of nanophotonic mode engineering with SAM avalanche architecture offers a scalable route toward fully integrated quantum photonic circuits with co‐located sources, detectors, and quantum processing elements.

## Author Contributions

**Kuldeep Singh:** investigation, writing – review and editing, formal analysis, data curation, writing – original draft, visualization, methodology. **Ekaterina Ponizovskaya Devine:** investigation, software, methodology, writing – review and editing, data curation, validation, formal analysis. **Md Sakibul Islam:** writing – review and editing, formal analysis, data curation. **Nitesh Singh:** writing – review and editing, formal analysis, data curation. **Wayesh Qarony:** conceptualization, supervision, funding acquisition, writing – original draft, writing – review and editing, project administration, resources, formal analysis, validation.

## Funding

This study was supported by the University of Central Florida.

## Supporting information


Supporting Information S1

## Data Availability

The data that support the findings of this study are available from the corresponding author upon reasonable request.
