# Supplementary material for: Nanophotonic Design of Germanium‐Silicon Single‐Photon Detectors for Integrated Quantum Photonic Circuits
Source: Nanophotonics. 2026 Jun 30;15(13):e70188. doi: 10.1002/nap2.70188 (PMC13642173; doi:10.1002/nap2.70188)
Supplement: Supplementary file 1 [file NAP2-15-e70188-s001.docx]

**Supplementary Information**

**Nanophotonic Design of Germanium-Silicon Single-Photon Detectors for Integrated Quantum Photonic Circuits**

*Kuldeep Singh^1^, Ekaterina Ponizovskaya Devine^1,2^, Md Sakibul Islam^1^, Nitesh Singh^1^, Wayesh Qarony^1,3,4,*^*

^1^Department of Electrical and Computer Engineering, University of Central Florida, Orlando, FL 32816, USA

^2^W&Wsens Devices Inc., 4546 El Camino, Los Altos, CA, USA

^3^Department of Physics, University of Central Florida, Orlando, FL 32816, USA

^4^CREOL, The College of Optics and Photonics, University of Central Florida, Orlando, FL 32816, USA

*Corresponding Email: [wayesh@ucf.edu](mailto:wayesh@ucf.edu)

This Supplementary Information provides detailed modeling, simulation methods, and extended results supporting the main manuscript.

**(I) Photonic Bandgap Design and Mode Confinement**

The photonic-crystal (PhC) geometry was optimized to engineer a photonic bandgap that overlaps with the telecom wavelength range, thereby enabling strong lateral confinement of the guided optical mode. As shown in Fig. S1(a), a hexagonal lattice of air holes is introduced in the silicon regions surrounding the Ge absorber, with rows of holes on each side. The lattice constant is selected such that the normalized frequency $a/\lambda\approx0.42$ lies within the telecom band, ensuring that the operating wavelengths fall inside the bandgap region.

The corresponding photonic band structure, calculated along the high-symmetry directions ($\Gamma$–X–M–$\Gamma$), is shown in Fig. S1(b). The black dashed curves represent the photonic band structure for TM polarization, while the red solid curves correspond to TE polarization, which exhibits a bandgap aligned with the target operating wavelength range. This bandgap suppresses in-plane propagation of optical modes within the patterned silicon regions, effectively creating lateral confinement analogous to a distributed Bragg reflector in the transverse direction.

As a result, the guided optical mode is strongly localized within the Ge absorption region while remaining evanescently coupled to the underlying silicon waveguide. This bandgap-assisted confinement enhances the overlap between the optical field and the Ge absorber while minimizing leakage into surrounding regions, thereby enabling efficient photon absorption within a compact device footprint. Importantly, the confinement mechanism is broadband and does not rely on high-Q resonant effects, making the design robust to fabrication variations and suitable for multi-wavelength quantum photonic applications.

**Fig. S1: Photonic-crystal geometry optimization and band structure engineering for telecom-band operation.** (a) Schematic of the nanophotonic Ge/Si waveguide incorporating photonic-crystal (PhC) patterning on both sides of the germanium absorption region. The PhC consists of a hexagonal lattice of air holes etched into the silicon regions, with rows of holes on each side of the Ge absorber. The lattice constant $a$is chosen such that $a\times\sqrt{3}=1091$ nm, corresponding to a periodicity engineered for operation in the telecom wavelength range. This geometry enables strong lateral optical confinement by introducing a photonic bandgap that suppresses mode leakage from the Ge absorber into the surrounding silicon. (b) Calculated photonic band structure of the optimized PhC along the high-symmetry directions $\Gamma$–X–M–$\Gamma$ in reciprocal space. The normalized frequency is expressed as $a/\lambda$, where $a$is the lattice constant and $\lambda$is the free-space wavelength. The black dashed curves represent the photonic band structure for TM polarization, while the red solid curves correspond to TE polarization, which exhibits a bandgap aligned with the target operating wavelength range. The shaded horizontal region near $\frac{a}{\lambda}\approx0.42$indicates the target operating frequency range corresponding to the telecom band (~1.3–1.55 µm). The optimization shifts and widens the photonic bandgap to overlap with this frequency range, enabling broadband confinement of guided modes within the Ge absorber.

**(II) Absorption and Quantum Efficiency**

The optical response of the Ge/Si SAM SPAD is evaluated using finite-difference time-domain (FDTD) simulations, which provide the spatial distribution of the electromagnetic field within the device. From the simulated electric field, both the optical absorption and the corresponding carrier generation within the germanium (Ge) region can be quantitatively determined.

The local optical absorption is governed by the imaginary component of the material permittivity. The volumetric power absorbed in the Ge layer is expressed as

$Q(x,y,z,\lambda)=\frac{1}{2}\omega\varepsilon_{0}\varepsilon^{''}\mid E(x,y,z,\lambda)\mid^{2}$	(1)

where $\varepsilon^{''}$is the imaginary part of the dielectric permittivity, $\varepsilon_{0}$is the vacuum permittivity, $\omega$ is the angular frequency of the optical field, and $E\left( x,y,z,\lambda\right)$is the electric-field amplitude. The complex permittivity is defined as $\varepsilon=(n-ik)^{2}$, where $n$ and $k$are the refractive index and extinction coefficient of Ge, respectively. The corresponding absorption coefficient is given by

$\alpha=\frac{4\pi k}{\lambda}.$	(2)
The total optical power absorbed within the Ge region is obtained by integrating the absorbed power density over the device volume. The internal quantum efficiency (IQE) is then calculated as

$IQE(\lambda)=\frac{1}{P_{\text{in}}}\iiint_{\text{Ge}} Q(x,y,z,\lambda)\text{ }dV,$	(3)
where $P_{\text{in}\text{ }}$is the optical power launched into the input waveguide. In this definition, only absorption occurring within the Ge layer contributes to the quantum efficiency, as this region is responsible for photocarrier generation.

Figure S2 presents the wavelength-dependent IQE for both planar and nanophotonic (photonic-crystal, PhC) Ge/Si SAM SPADs with Ge absorber lengths of 4 µm, 8 µm, 12 µm, and 16 µm. The nanophotonic design consistently exhibits significantly higher IQE compared to the planar geometry across the telecom wavelength range. This enhancement arises from photonic-crystal–induced mode confinement, which increases the overlap of the optical field with the Ge absorber while suppressing leakage into the surrounding regions.

**Fig. S2: Wavelength-dependent internal quantum efficiency of planar and nanophotonic Ge/Si SAM SPADs.** Simulated internal quantum efficiency (IQE) as a function of wavelength for both planar and nanophotonic (photonic-crystal, PhC) Ge/Si separate-absorption–multiplication (SAM) SPADs with Ge absorber lengths of 4 µm, 8 µm, 12 µm, and 16 µm. Solid curves represent the nanophotonic (PhC) design, while dashed curves correspond to the planar geometry.

For both planar and nanophotonic devices, the IQE increases with Ge absorber length due to the longer interaction length between the guided optical mode and the absorbing material. However, the improvement is substantially more pronounced in the nanophotonic architecture, where strong optical confinement enables near-unity quantum efficiency even for relatively compact device dimensions. In particular, the photonic-crystal design with a 16 µm Ge absorber approaches unity IQE over a broad spectral range, demonstrating the effectiveness of nanophotonic mode engineering in enhancing absorption without relying on resonant effects.

**(III) Avalanche Gain and Breakdown Characteristics**

Fig. S3(a) shows the device cross-section, dimensions, and doping concentrations used in the electrostatic-field and avalanche-gain simulations. Figure S3(b) presents the simulated avalanche gain as a function of reverse-bias voltage for the planar Ge/Si separate-absorption–multiplication (SAM) SPAD, obtained from carrier-transport and impact-ionization simulations. At low reverse bias, the electric field in the intrinsic silicon multiplication region is insufficient to sustain significant impact ionization, resulting in modest gain. As the reverse bias increases, carrier multiplication becomes progressively stronger due to the increasing electrostatic field within the silicon multiplication layer.

**Fig. S3: Simulated avalanche gain versus reverse-bias voltage in planar and nanophotonic Ge/Si SPADs.** (a) Device cross-section showing the dimensions and doping concentrations used in the electrostatic-field and avalanche-gain simulations. (b) Simulated avalanche gain as a function of reverse-bias voltage for the planar Ge/Si separate-absorption–multiplication (SAM) SPAD. The gain increases gradually with bias and exhibits a sharp rise near the breakdown voltage (~28 V), indicating the onset of Geiger-mode operation. (c) Simulated avalanche gain as a function of reverse-bias voltage for the nanophotonic Ge/Si SAM SPAD. Owing to enhanced electrostatic-field confinement, breakdown occurs at a lower voltage (~18 V), resulting in an earlier transition to high-gain Geiger-mode operation.

As the planar device approaches the simulated breakdown voltage (~28–30 V), the ionization rate increases rapidly, producing a sharp transition from linear-mode amplification to Geiger-mode operation and gain values exceeding 10^5^. The SAM architecture spatially confines avalanche multiplication to intrinsic silicon while maintaining the Ge absorber outside the peak-field region. This separation minimizes the influence of Ge-related defects and trap-assisted carrier generation on the avalanche process.

Figure S3(c) shows the corresponding simulated avalanche gain for the nanophotonic Ge/Si SAM SPAD. Owing to enhanced electrostatic-field confinement in the silicon multiplication region, the nanophotonic design reaches avalanche breakdown at a lower simulated voltage (~18 V), enabling an earlier transition to high-gain Geiger-mode operation. These results indicate that nanophotonic and electrostatic-field co-design can support efficient avalanche multiplication while preserving the separate-absorption–multiplication functionality required for low-noise operation.

The simulated reverse-bias dark-current characteristics of the planar Ge/Si SAM SPAD are shown in Fig. S4. The dark current remains low at moderate reverse-bias voltages and increases rapidly as the device approaches avalanche breakdown (~30 V). This behavior is consistent with enhanced field-assisted carrier generation and the onset of avalanche multiplication in the silicon multiplication region. Together, the gain and dark-current simulations provide a self-consistent indication of the breakdown behavior predicted for the proposed SAM architecture.

**Fig. S4:** Simulated dark-current–voltage (I–V) characteristics of the planar Ge/Si SAM SPAD under reverse-bias operation. The dark current remains low at moderate reverse-bias voltages and increases rapidly as the device approaches avalanche breakdown (~30 V). This behavior is attributed to enhanced field-assisted carrier generation and the onset of avalanche multiplication in the silicon multiplication region. The results are consistent with the simulated avalanche-gain characteristics shown in Fig. S3.

**(IV) Trade Off Between Internal Quantum Efficiency (IQE) and Dark Current**

Table S1: This table presents the comparative analysis between the Internal quantum efficiency and dark current as the Ge length increases.

| **Length of Ge (µm)** | **IQE (%)** | **Dark Current (nA)** |
| --- | --- | --- |
| 4 | 76 | 0.3 |
| 8 | 89 | 0.6 |
| 12 | 95 | 0.9 |
| 16 | 99 | 1.2 |
